# Gut microbiota in ischemic stroke: Where we stand and challenges ahead

**DOI:** 10.3389/fnut.2022.1008514

**Published:** 2022-12-01

**Authors:** Jiaxin Long, Jinlong Wang, Yang Li, Shuai Chen

**Affiliations:** ^1^Key Laboratory of Systems Health Science of Zhejiang Province, School of Life Science, Hangzhou Institute for Advanced Study, University of Chinese Academy of Sciences, Hangzhou, China; ^2^College of Pharmacy, Hunan University of Chinese Medicine, Changsha, China

**Keywords:** gut microbiota, microbial metabolites, ischemic stroke, oxidative stress, inflammation, apoptosis

## Abstract

Gut microbiota is increasingly recognized to affect host health and disease, including ischemic stroke (IS). Here, we systematically review the current understanding linking gut microbiota as well as the associated metabolites to the pathogenesis of IS (e.g., oxidative stress, apoptosis, and neuroinflammation). Of relevance, we highlight that the implications of gut microbiota-dependent intervention could be harnessed in orchestrating IS.

## Introduction

Stroke ranks among the top three fatal diseases in developed countries and has even more lethality in developing countries (1, 2). The stroke is classified to ischemic stroke (IS) and hemorrhagic stroke. The IS is responsible for 87% of stroke (3). Except for population growth and aging, non-negligible risk factors such as high body mass index, environmental particulate pollution, high fasting blood glucose, high systolic blood pressure, alcohol consumption, low physical activity, renal insufficiency, and high temperature also exacerbate the prevalence of IS (4). Clinically, thrombolysis is an early treatment for IS. Recombinant tissue plasminogen activator (rtPA), an effective thrombolytic drug, is the only Food and Drug Administration (FDA)-approved medicine for acute IS. However, the bleeding risk and short treatment window (3–4.5 h after onset) of rtPA limits its clinical use (5). Considering that postischemic neuroprotective therapy can prolong the time window of thrombolytic treatment, it is urgent to develop neuroprotective strategies to protect brain cells from ischemia and reperfusion injury (6–8).

Gut microbiota enables bidirectional communication between the gut and the brain through the “microbial-gut-brain axis”, associated with immune, endocrine, and neuromodulatory mechanisms (9, 10). Cytokines and chemokines produced in the brain could be released into the systemic circulation after IS, potentially impacting gut microbiota imbalance. Clinical studies showed that the diversity of gut microbiota in the IS population is significantly reduced, among which *Escherichia, Megamonas, Bacillus, Bifidobacterium*, and *Ruminococcus* are increased significantly, while *Parabacteroides, Ekmanella, Prevotella*, and *Faecalibacterium* burden are decreased in IS population compared to the healthy people (11, 12). Likewise, Yamashiro et al. studied the intestinal microbiota of 41 IS patients and 40 healthy people and confirmed the significant differences of gut microbiota between them. In the middle cerebral artery occlusion (MCAO) mouse model, the gut microbiota could cross the intestinal barrier and migrate to various organs through the blood to induce inflammatory responses (13, 14). These findings suggest that gut microbiota might be used as a neuroprotective strategy for IS (15, 16). Interestingly, studies on animal experiments demonstrated that fecal microbiota transplantation from healthy donors could restore the gut microbiota imbalance caused by IS and consequently improved neural function (17). Moreover, gut microbial metabolites were found to regulate the IS process. For example, trimethylamine oxide (TMAO), a metabolite of Gammaproteobacteria, can promote atherosclerosis and cerebral vascular embolism by affecting cholesterol reverse transport and metabolism (18). The immunogenic endotoxin lipopolysaccharide (LPS) may promote neuroinflammation by inducing the migration of peripheral immune cells to the brain (19). Short-chain fatty acids (SCFAs) can reduce the severity of neuroinflammation, protect brain tissue, and reduce neuronal damage in IS patients (17).

This review aims to elucidate the mechanism of gut microbiota in the pathological process of IS and provide new ideas for IS treatment.

## IS pathogenesis and microbiota

**Figure 1:**
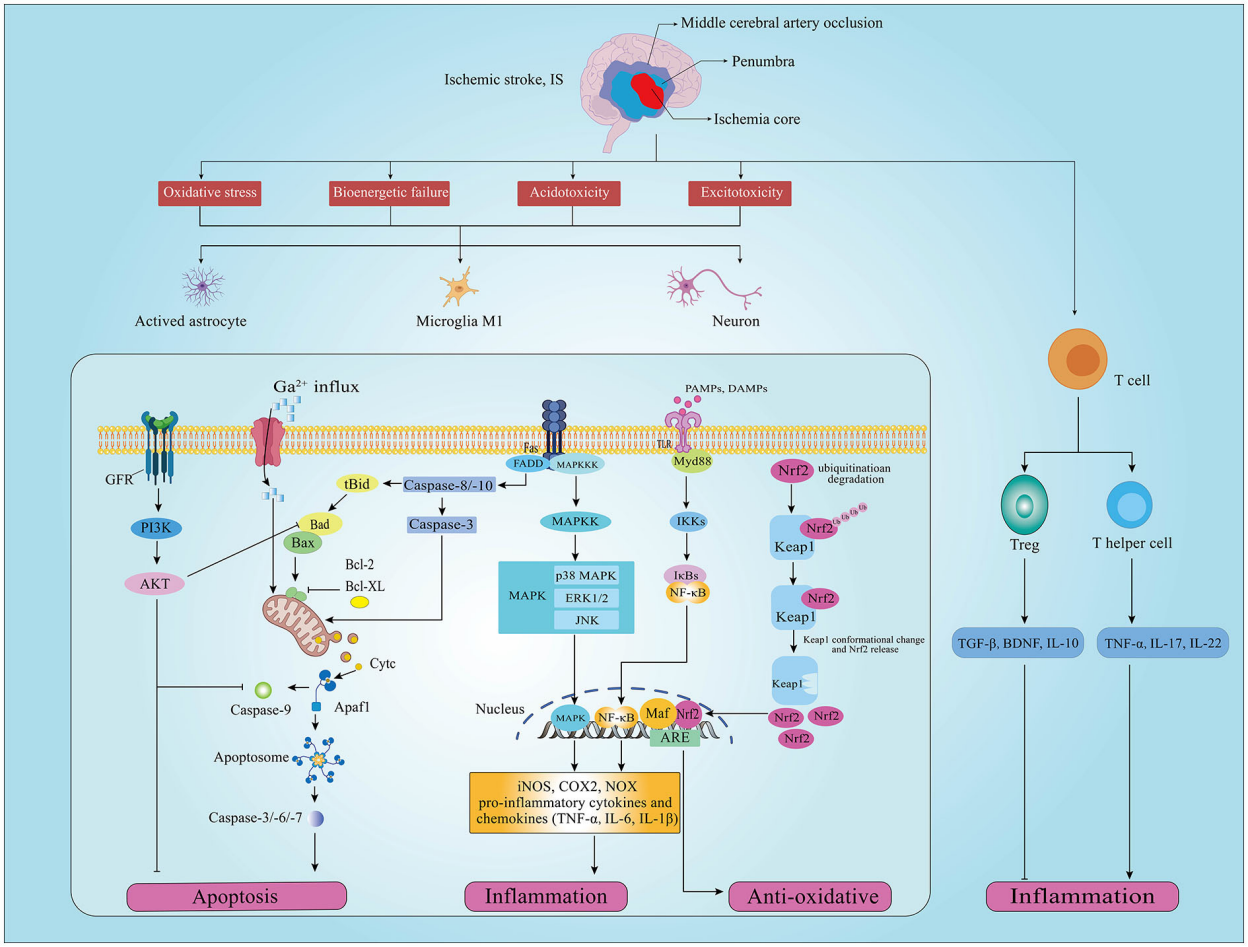
Schematic diagram of oxidative stress, apoptosis, and inflammation signaling pathways involved in ischemic stroke (IS). Oxidative stress, inflammation and apoptosis occur in neurons of the penumbra after IS, which are regulated by different upstream pathways. (GFR, growth factor receptor; PI3K, phosphatidylinositol 3-kinase; AKT, protein kinase B; Bad, Bcl2 associated agonist of cell death; Bcl-2, B-cell lymphoma-2; Bcl-XL, B-cell lymphoma-extra large; Bax, Bcl-2-associated X; Cytc, cytochrome c; Apafl, apoptotic protease activating factor-1; TGF-β, transforming growth factor beta; BDNF, brain-derived neurotrophic factor; Keapl, Kelch-like ECH-associated protein 1; Nrf2, nuclear factor E2 related factor 2; Ub, ubiquitination; ARE, adenylate Uridylate-rich element; Maf, macrophage activating factor; TLR4, toll-like receptor 4; Myd88, myeloid differentiation primary response 88; Ikks, I-κB kinase; NF-κB, nuclear factor kappa B; IL-1β, interleukin 1 beta; MAPK, mitogen-activated protein kinases; JNK, c-Jun N-terminal kinase; ERK, extracellular regulated protein kinases; iNOS, inducible NO synthase; COX2, cyclooxygenase 2, COX2; NOX, nitrogen oxide; TNF-α, tumor necrosis factor alpha; Treg, regulatory T cell).

IS is associated with the interruption of cerebral blood flow induced by thrombotic or embolic occlusion of a cerebral artery. The pathological change of brain cell damage in IS is a dynamic developmental process of complex spatiotemporal cascade reactions in the penumbra area involving oxidative stress, inflammation, and apoptosis (20–22). Acute reduction or deprivation of cerebral blood flow limits glucose and oxygen access to brain tissue, and the limitation of glucose and oxygen reduces adenosine triphosphate (ATP) production and further induces bioenergetic failure, acidotoxicity, and excitotoxicity in brain cells (23). ATP consumption also causes the depolarization of neuronal plasma membrane and dysfunction of ATP-dependent ion pumps, further disrupting sodium, potassium, and calcium (Ca) ionic balance and leading to anoxic depolarization in neurons and glial cells (23, 24). Neuronal cell bioenergetic failure and depolarization lead to cellular sodium and Ca^2+^ accumulation, inducing mitochondria dysfunction and catabolic enzyme activation to promote reactive oxygen species (ROS) and oxidative intermediates generation, resulting in endogenous redox disbalance and oxidative severe stress damage (cell apoptosis and necrosis) (25). Furthermore, complex triggers such as bioenergetic failure, ROS, and cell necrosis initiate brain cell immune response characterized by the activation of glial cells, the infiltration of monocytes/macrophage and leukocytes, and the production of pro-inflammatory cytokines and chemokines (26) (Figure 1).

**Figure 2:**
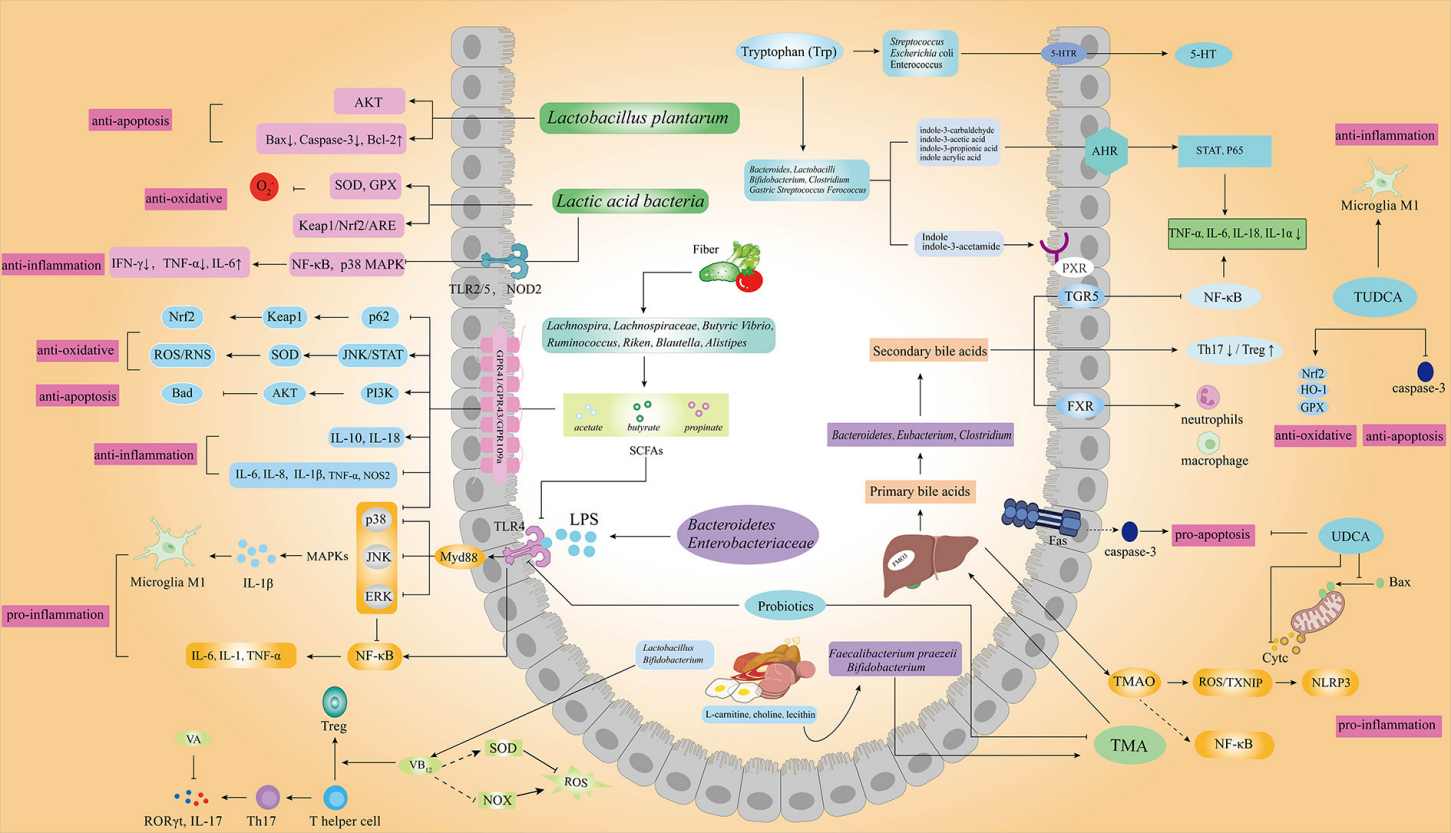
Gut microbiota and its metabolic products associated with IS. Gut microbiota and the metabolism of SCFAs, tryptophan, bile acids, TMAO, LPS, and vitamins play important roles in oxidative stress, inflammation, and apoptosis of IS through different signaling pathways. (AKT, protein kinase B; Bax, Bcl2-associated X; Bcl-2, B-cell lymphoma-2; SOD, superoxide dismutase; GPX, glutathione peroxidase; Keapl, kelch-like ECH-associated protein 1; Nrf2, nuclear factor E2 related factor 2; Ub, ubiquitination; ARE, adenylate uridylate-rich element; IFN-γ, interferon gamma; IL-6, interleukin 6; IL-17, interleukin 17; IL-18, interleukin 18; IL-1α, interleukin 1 alpha; Treg, regulatory T cell; Th17, helper T 17 cell; RORγt, retinoic acid related orphan nuclear receptor γt; IL-10, interleukin 10; iNOS, inducible NO synthase; COX2, cyclooxygenase 2; NOX, nitrogen oxide; NOS2, nitric oxide synthase 2; GPR43/GPR41, G protein-coupled receptors 43/41; MAPK, mitogen-activated protein kinases; JNK, c-Jun N-terminal kinase; ERK, extracellular regulated protein kinases; TMA, trimethylamine; TMAO, trimethylamine oxide; TXNIP, thioredoxin-interacting protein; FMO3, flavin-containing monooxygenase 3; FXR, farnesoid X receptor; TNF-α, tumor necrosis factor alpha; PI3K, phosphatidylinositol 3-kinase; STAT, signal transducer and activator of transcription; TUDCA, tauroursodeoxycholic acid; UDCA, ursodeoxycholic acid; HO-1, heme oxygenase-1; PXR, pregnane X receptors; AHR, aryl hydrocarbon receptors; 5-HT, 5-hydroxytryptamine; 5-HTR, 5-hydroxytryptamine receptor).

Numerous studies have reported the role of gut microbiota in oxidative stress, inflammation, and apoptosis (Figure 2), indicating its potential protective effects in IS (27–30).

### Oxidative stress and microbiota

Oxidative stress (OS) induced by the excess generation of ROS attacks cellular lipids, proteins, and nucleic acid, causing cell apoptosis, death, or even necrosis (25). OS also promotes the release of cytochrome C (Cytc) and the activation of astrocytes and microglia, inducing early inflammation and cell apoptosis (31). Of note, the transcription factor NF-E2-related factor (Nrf2)/antioxidant response element (ARE) signaling pathway is recognized as the central player in the antioxidant system (32). By binding to its inhibitor Kelch-like epichlorohydrin-associated protein-1 (Keap1) under normal conditions, Nrf2 mainly exists in the cytoplasm as an inactive state but would be rapidly degraded following ubiquitin protease activation, maintaining the regular and low transcriptional activity of Nrf2 (33). When free sulfhydryl groups (i.e., Nrf2 or electrophilic stress sensors) are oxidized by ROS, Nrf2 is activated and translocated into the nucleus to interact with AREs, resulting in the gene expression of phase II antioxidants and detoxification enzymes (Figure 1).

The gut microbiota can affect the ROS level. Both commensal and pathogenic bacteria in the gut can alter intracellular ROS by modulating mitochondrial activity, which may be related to Keap1/Nrf2/ARE signaling pathway (34, 35) (Figure 1). For example, some Lactobacilli can stimulate cells to produce ROS through specific membrane components or secreted factors (36); in addition, LPS from gram-negative bacteria can increase the sensitivity of neurons to OS (37). Indeed, IS and microbiota dysbiosis often occurs simultaneously in patients and animal models (38, 39). Interestingly, ROS generated in IS stimulates IL-6 and other inflammatory factors production by activating the nuclear factor κB (NF-κB) pathway, indirectly increasing the abundance of facultative anaerobic bacteria (mainly Enterobacteriaceae) and promoting microbiota dysbiosis (40, 41). These studies suggest that targeting gut microbiota is an effective method to regulate OS and further prevent IS.

### Apoptosis and microbiota

Neurons and glial cells in the penumbra undergo apoptosis in the hours to days following IS (42). Apoptosis is an ATP-dependent programmed cell death characterized by the early degradation of DNA and intact cell membrane without cellular contents efflux. Ca^2+^ overload and mitochondrial dysfunction in IS are the main inducers of neuronal apoptosis, which consist of two principal pathways: the endogenous apoptosis (or mitochondrial) pathway and the exogenous apoptosis (or death receptor) pathways (43). The endogenous apoptosis pathway is induced by the release of key apoptosis-related proteins in mitochondria, and the exogenous apoptosis pathway is triggered by the combination of surface death receptors and their ligands (43) (Figure 1).

Apoptosis stimuli such as Ca^2+^ overload and ROS mediate endogenous apoptosis. Excessive Ca^2+^ ions influx at the pre-synaptic terminal causes uncontrolled glutamate release to the synaptic terminal, activating ionotropic N-methyl-d-aspartate receptors (NMDAR) and resulting in Ca^2+^ further influx (44). Abnormal aggregation of Ca^2+^ leads to calpain activation to split the Bcl-2 interaction domain (BID) into truncated BID (tBID), together with caspase-8 cleavage. tBID interacts with the Bad-Bax for opening the mitochondrial transition pore (MTP) and promoting the release of mitochondrial Cytc, endonuclease G, and apoptosis-inducing factors (AIFs) (45, 46). Cytc binds to the apoptotic protease activating factor 1 (Apaf-1) and pro-caspase-9 to form apoptosomes, activating caspase-9 and inducing downstream effector caspase-3. Activation of caspase-3 leads to mitochondrial membrane permeabilization, chromatin condensation, DNA fragmentation, and ultimately cell death (47). Bax, Bad, and BH3 protein polymers can bind on the outer membrane of mitochondria to promote MTP formation, while Bcl-2 family inhibitors could inhibit MTP formation by competitively binding with Bax and Bad (48). The exogenous apoptosis pathway is activated by the binding of cell surface death receptors such as TNF receptor 1 (TNFR-1), Fas receptor (FasR), p75 neurotrophin receptor (p75NTR), and their corresponding ligands (49). The combination of legends, such as TNF-α and Fas ligand (FasL), and death receptors leads to the activation of caspase-8 and caspase-10, which in turn activate downstream effector, finally leading to neuronal death (50, 51). Although reversing the necrotic neurons in the infarcted area is difficult, targeted interfering upstream apoptosis signals is a potential neuroprotective strategy to rescue the damaged neurons (52) (Figure 1).

Gut microbiota is reported to regulate the apoptosis of multiple cells from the intestine, kidney, and brain (53, 54). Liu et al. demonstrated that the intestinal probiotic *Clostridium butyricum* could phosphorylate protein kinase B (AKT) by regulating the protein expression levels of brain-derived neurotrophic factor (BDNF), Bcl-2, and Bax to prevent neuronal apoptosis in cerebral I/R injury diabetic mice (55). SCFAs are found reduced in IS mice model, within which the butyric acid showed the highest negative correlation with IS parameter (56). Ursodeoxycholic acid (UDCA) and taurine deoxycholic acid (TUDCA) could be produced by microbiota and are also reported to play important roles in regulating neuronal apoptosis (57, 58). Collectively, these studies indicate that gut microbiota could be a potential strategy for targeting apoptosis in IS therapy.

### Inflammation and microbiota

The activation of inflammatory cells, like microglia, astrocyte, and T cell, is the hallmark of the inflammatory response in IS (59). After IS onset, insufficient oxygen, glucose, and energy production result in the accumulation of some toxic metabolites, such as excitotoxic products, acidic metabolites, and inflammatory mediators, and cause extensive neuron and glial cell death. These dead cells induce innate immune inflammatory responses by releasing damage-associated molecular patterns (DAMPs) [e.g., ATP, high mobility histone B (HMGB1), and hypoxia-inducible factor 1α (HIF-1α)] (23). Indeed, microglia activation releases danger signals to activate the innate immune response in lesions, promoting tumor necrosis factor-alpha (TNF-α), interleukin-1β (IL-1β), interleukin-6 (IL-6), and other potentially cytotoxic molecules such as prostaglandins, ROS, and nitric oxide (NO) (60). Activated astrocytes also release inflammatory factors, such as inducible nitric oxide synthase (iNOS), cyclooxygenase-2 (COX2), and NADPH oxidase (NOX) (61). Cytokines produced by microglia and astrocytes play critical roles in IS pathology (62). Proinflammatory cytokines (e.g., TNF-α, IL-1β, and IL-6) and chemokines exacerbate brain damage, while anti-inflammatory cytokines [e.g., IL-10 and transforming growth factor beta (TGF-β)] are neuroprotective (63, 64) (Figure 1).

Toll-like receptors (TLRs) expressed in astrocytes and microglias play essential roles in DAMPs/pathogen-associated molecular pattern (PAMPs) recognization and inflammatory responses (65). For example, TLR4 activated by DAMPs through the downstream myeloid differentiation factor 88 (MyD88)-dependent pathway can mediate NF-κB translocation to the nucleus for initiating the transcription of inflammatory cytokines and the following inflammatory response cascades, thereby inducing vascular damage (66). Mitogen-activated protein kinases (MAPKs) is also the critical signaling molecules associated with inflammatory responses by regulating multiple extracellular kinases, including extracellular signal-regulated protein kinase (ERK)1/2, p38MAP Kinase (p38), c-Jun N-terminal kinases (JNK) (67). MAPK signaling pathway affects the balance of pro-inflammatory and anti-inflammatory responses by regulating the production of TNF-α, IL-1, IL-6, and IL-12 (68). Moreover, T cells are recruited to the injured brain tissue within the first days after stroke and play a decisive role in secondary neuroinflammation in IS (69–71). The helper T (Th) cell subpopulations have differential effects on IS outcomes. Pro-inflammatory such as Th1 and Th17 promote inflammatory damage, worsing IS outcomes, whereas regulatory T cells (Tregs) have been shown to suppress an excess neuroinflammatory reaction to brain injury (72, 73) (Figure 1). Therefore, it could effectively alleviate the inflammation injury of IS by targeting the inflammation-related cells and cytokines.

Gut microbiota could affect the central neuron system (CNS) and brain function by interacting with the immune system (74). IS is associated with decreased SCFAs and SCFAs-producing bacteria such as Bacteroidaceae, Ruminococcaceae, and *Faecalibacterium* and also associated with increased fecal valeric acid, which is positively correlated with inflammatory marker levels (e.g., high-sensitivity C-reactive protein and white blood cell counts) (38, 75, 76). Numerous studies have demonstrated gut microbiota is a key regulator of IS-related immune cells (77, 78). For example, *Group B Streptococcus* and *Listeria monocytogenes* could stimulate the differentiation of effector T cell, increase T cell infiltration to the brain, and disrupt the integrity of the blood-brain barrier (BBB), consequently inducing neuroimmune inflammatory responses (79). The gut microbiota is also associated with inflammation-associated mediators. Germ-free (GF) mice recolonized with microbiota from IS mice showed higher IL-17 and IFN-γ expression and Th17 cell infiltration in hemispheres in the context of IS, than those received the health mouse microbiota (15). Moreover, in the experimental IS mouse model, antibiotics-induced “GF” mice show moderate brain injury than those mice with normal microbiota (80). Together, it is reasonable that gut microbiota may be a key target for the effective management and treatment of IS therapy.

## Microbiota, microbial metabolites, and IS

### Probiotics

Probiotics such as *Lactobacillus, Bacillus subtilis*, and *Bifidobacterium* have positive effects on anti-oxidative, immune modulation, and pathogen antagonism (81). Probiotic fermented milk showed significant antioxidant capacity *in vitro* and *vivo* as a dietary source (82, 83). Lactic acid bacteria (LAB) could be autolyzed under stress, such as low pH conditions, and release intracellular antioxidant substances *in vitro* (84). I*n vivo*, LAB like *Lactobacillus plantarum* was reported to have salutary effects against exogenous insults *via* the Nrf2/ARE signaling pathway in mice and drosophila (85). Cheon et al. found that LAB KU200793 in fermented foods showed high antioxidant activity and protected human neuroblastoma cell SH-SY5Y from 1-methyl-4-phenyl pyridine-induced damage (86). The modulatory role of probiotics in immunity and inflammation has long been investigated, although the precise mechanisms are still not fully understood (87, 88).

In terms of apoptosis, Sirin, S. et al. found that *Bulgarian bacterium B3* and *Lactobacillus plantarum GD2* protected amyloid beta-induced mitochondrial dysfunction SH-SY5Y cells from apoptosis (89). *In vivo* experiments showed that probiotic preparations (*Lactobacillus helveticus R0052* and *Bifidobacterium longum R0175*) attenuated LPS-induced apoptosis in rat hippocampus through the gut-brain axis by increasing Bcl-2 and inhibiting Bax and caspase-3 (90). Probiotic cocktail, including bifidobacteria, *lactobacillus, laccoccus*, and yeast, showed anti-apoptosis function by activating the AKT signaling pathway of the hippocampus (91). The evidence supports the beneficial effect of probiotics on IS therapy.

Moreover, LAB may trigger natural killer T cells to promote IFN-γ (92) and alleviates intestinal inflammation by regulating the NF-κB and p38 MAPK pathway mediated by TLR2/TLR5 receptors (93) and NOD2 receptors (94). The mechanism might be associated with LAB-secreted polypeptide, which could inhibit I-κB phosphorylation and the process of NF-κB translocation to the nucleus (95). Studies have also demonstrated the role of microbiota in neuronal function, such as neurogenesis and neuroinflammation (96). Indeed, *Lactobacillus plantarum* showed significant anti-inflammation activity by decreasing IFN-γ, TNF-α, and BBB permeability and increasing IL-10 (97, 98). Other probiotics, such as *Faecalibacterium prausnitzii*, also had anti-inflammatory functions in depression-like and anxiety-like behavior rat model (99) (Figure 2).

### Short-chain fatty acids

SCFAs are gut microbiota metabolites that could serve as key signaling molecules in the gut-brain communication (100) and can control neurodevelopment, neurotransmitters, and microglia activation (101, 102). Acetic acid, propionic acid, and butyric acid account for more than 95% of intestinal SCFAs and play important roles in oxidative stress, immune inflammation, and neuronal apoptosis (103). SCFAs, especially butyrate, can regulate oxidative stress. In healthy humans, administration of butyrate at physiological concentrations has been shown to increase the antioxidant glutathione and reduce ROS production (104, 105). Wang et al. showed that sodium butyrate supplementation relieves cerebral ischemia-reperfusion injury *via* increasing superoxide dismutase expression in mice, which was related to the JNK/STAT pathway (106). Also, sodium butyrate could improve brain damage in mice by reducing the oxidative stress of neurons *via* the Keap1/Nrf2/HO-1 pathway (99). Butyrate-producing *Clostridium butyricum* resists oxidative damage by regulating the p62-Keap1-Nrf2 signaling pathway (107).

The regulatory role of SCFAs on cell apoptosis mainly focuses on the intestinal cell (108–110). Some studies reveal the anti-apoptosis role of SCFAs in IS. For example, butyrate was reported against neuronal apoptosis *via* up-regulating histone H3 acetylation, heat shock protein 70 (HSP70), and p-Akt and down-regulating p53 in ischemic brain tissue (111, 112). SCFAs play important roles in regulating neuroinflammation and microglia and could reduce inflammation after IS (113). Oral administration of SCFAs can repair the defective microglia, promote the activation of the anti-inflammatory phenotype of microglia, and increase the expression of tight junction protein to enhance the structural integrity of BBB while reduce the harmful substances invasion of the brain (114, 115). Acetate reduces LPS-induced glial cell activation by activating GPR43 and inhibiting IL-1β expression and MAPK phosphorylation in rats with neuroinflammation (116, 117). Patiala et al. found that sodium butyrate down-regulates pro-inflammatory mediators TNF-α and NOS2 and up-regulates the expression of anti-inflammatory mediator IL-10 in the MCAO mice (118) (Figure 2).

### Tryptophan and its derivatives

The level of serum L-Trp in IS patients is increased significantly, suggesting that Trp may be a potential biomarker of IS (119). Wang et al. reported that Trp supplementation affected immunity by regulating the balance of anti-inflammatory cytokines and pro-inflammatory cytokines and signal transducer and activator of transcription 3 (STAT3) and p65 signal transduction (120). 5-hydroxytryptamine (5-HT), the vital metabolite of Trp, serves as the junction of the gut-brain microbiota axis and the suppressor of inflammation (121). Also, Trp can produce indole and indole derivatives (e.g., indole-3-propionic acid), the ligands of pregnane X receptors (PXR) and aryl hydrocarbon receptors (AHR), *via* the gut microbial, such as *Lactobacillus, Clostridium*, and *Peptostreptococcus* (122–125). Rothhammer et al. found that indole-3-acetic acid can regulate the inflammatory response of the central nervous system, which might be associated with inhibiting the secretion of pro-inflammatory factors such as TNF-α, IL-1β, and monocyte chemoattractant protein-1 (MCP-1) from macrophages (126, 127). Indole-3-acetaldehyde produced by *Lactobacillus reuteri* can increase the expression level of IL-22 (127) (Figure 2). In conclusion, the function of Trp metabolism for regulating inflammation may provide a therapeutic solution for IS.

### Bile acids

BA metabolites are one of the most widely metabolites in host-microbe interactions and may play critical roles in the oxidative stress, apoptosis, and inflammation of IS. Gut microbiota regulates BA synthesis by regulating enzyme Cholesterol 7α-hydroxylase (CYP7A1), CYP7B1, and CYP27A1 and in turn, BA could re-modulate gut microbiota by promoting BA metabolism-microbiota growth and prevent the other microbes (18). For example, *Clostridium* affects the metabolism of BA, inhibits the activation of farnesoid X receptor (FXR), and promotes inflammation (128).

Indeed, a 20-year prospective follow-up study showed decreased BA excretion functioned as an independent risk factor for IS incidence and mortality (129). A clinical study found that total BAs levels were inversely associated with 3-month mortality in patients with acute IS (130). The reason may be partly associated with immunity because the imbalance of Th17/Treg was reported to aggravate IS (131), while BAs could control the host immune response by directly regulating the balance of the Th17/Treg (132, 133). Studies have shown that gut microbiota could affect the occurrence and development of IS *via* metabolizing primary BAs produced by the liver to secondary BAs by *Bacteroides, Eubacterium*, and *Clostridium spp*. (134, 135). In recent years, *Nature* has reported that UDCA, an intestinal bacteria-derived secondary bile acid, showed anti-oxidative activity by reducing the production of ROS and reactive nitrogen production and maintaining the level of GSH (136). UDCA was also reported had an anti-apoptotic activates by maintaining mitochondrial membrane integrity through preventing the transfer of apoptotic Bax from the cytoplasm to mitochondria, the release of Cytc, and the subsequent activation of caspase (57, 137, 138). Furthermore, UDCA could also prevent apoptosis through the Fas-L-induced death receptor pathway (139). TUDCA, a taurine conjugate of UDCA that could pass the BBB (140, 141), was reported to inhibit oxidative stress by enhancing the expression of Nrf2, the antioxidant enzymes heme oxygenase-1(HO-1), and glutathione peroxidase (GPx) (142). TUDCA also plays an anti-inflammatory role by inhibiting the activation of glial cells (143). Rodrigues reported that TUDCA could significantly increase the level of brain bile acid, inhibit mitochondrial swelling and caspase-3 expression, reduce infarct area, and improve neural function (144). Hence, TUDCA showed a neuroprotective effect against apoptosis in the MCAO animal model; the mechanism may be associated with activating PI3K dependent Bad signaling pathway and inhibiting E2F-1/p53/Bax pathway (145–148) (Figure 2). In conclusion, targeting BAs could also be one of the neuroprotective strategies of IS.

### Choline metabolites

Choline metabolites mainly include trimethylamine (TMA), dimethylamine, methylamine, dimethylglycine, and other substances. After being absorbed into the blood, TMA is rapidly oxidized to TMAO by liver flavin-containing monooxygenase-3 (FMO-3) or other flavin monooxygenases (FMOs) (149). TMAO could also be produced by microbiota such as *Faecalibacterium praezeii* and *Bifidobacterium* (150). Studies showed that TMAO could accelerate the process of atherosclerosis by enhancing platelet aggregation, inflammation, and oxidative stress (151, 152). Li et al. found that a high concentration of TMAO could promote the expression of TNF-α, IL-1β, and superoxide, inhibit the expression of endothelial nitric oxide synthase (eNOS), and cause vascular inflammation and oxidative stress (153). Mechanistically, TMAO induces oxidative stress and inflammation response *via* the ROS/thioredoxin-interacting protein (TXNIP)/ NOD-, LRR- and pyrin domain-containing protein 3 (NLRP3) signaling pathway (154, 155). Besides, TMAO induces foam cell formation through MAPK/JNK pathway for promoting arterial occlusion (156). Glial scarring and astrogliosis models are widely used to mimic the clinical conditions of the human stroke (157). Recently, it has been demonstrated that TMAO promoted reactive astrogliosis and glial scarring by interacting with the Smad ubiquitination-related factor 2 (Smurf2)/Activity-like kinase 5 (ALK5) pathway, resulting in severer nerve injury after IS (158). Reducing TMAO and its precursor TMA is an important strategy to prevent IS. Some studies have shown that probiotics could reduce plasma TMAO levels and alleviate related diseases. Qiu et al. found *Enterobacter aerogenes* reduced serum TMAO and cecal TMA levels in mice with a high-choline diet (159). And they subsequently found that *Lactobacillus plantarum* could modulate cecal TMA or serum TMAO levels by affecting gut microbiota but not the expression of liver FMO3, which could stimulate the reverse transport of cholesterol against inflammation, finally alleviating atherosclerosis caused by TMAO (160) (Figure 2). These studies indicate promising strategies for preventing and treating IS by reducing the intake of choline-rich diet or using probiotics to inhibit the formation of TMAO.

### Lipopolysaccharide

LPS, also known as endotoxin, is present in the outer membrane of Gram-negative bacteria, such as Bacteroidetes and Enterobacteriaceae (161–163). Bacteroides account for approximately 50% of the human gut microbiota (164) and produce a considerable amount of LPS (165, 166). Lehr HA et al. found LPS increased atherosclerosis in hypercholesterolemic and decreased atherosclerosis and plaque in mice lacking TLR4 (167). Clinical research shows that LPS increases the risk of IS, and elevated plasma LPS levels are associated with poorer short-term outcomes of acute IS patients (168). Indeed, LPS significantly increased the infarct volume in MCAO mice, partially *via* TLR4/NF-κB pathway, resulting in the production of pro-inflammatory cytokines such as TNF-α, IL-1, and IL-6 (169). LPS aggravated brain damage, while increasing *Lactobacillus, Bifidobacteria*, and butyric acid-producing bacteria were reported to inhibit TLR4 signaling, reduce plasma LPS level, as well as and maintain T cell abundance (for example, reducing Th1 and Th17 cells while increasing Treg cells) (Figure 2).

### Vitamins and related compounds

Vitamins, including vitamin A (VA), vitamin B (VB), vitamin K (VK), vitamin H (VH), folic acid, thiamine, and riboflavin, are essential micronutrients that cannot be synthesized in mammals and must be absorbed through the intestine. The gut microbiota is a great source of crucial vitamins. *Lactobacillus* and *Bifidobacterium* are inseparable from VB, VK, and folic acid synthesis (170). VA was reported to regulate intestinal immune response and can maintain gut microbiota homeostasis (171, 172). Studies have shown that VA supplementation can relieve arterial occlusion, which is related to the role of VA in reducing IL-17 and RORγt gene expression and Th17 cells (173, 174). As an essential cofactor for energy production, VB directly participates in the tricarboxylic acid cycle of cells, promotes ATP production, and provides energy for exercise. VB_12_ is an essential cofactor in amino acid and nucleotide biosynthesis in gut microbiota. It protects against IS by scavenging free radicals to inhibit oxidation-induced damage and resist oxidation neuroinflammation and apoptosis (175, 176). VB_12_ can also change gut immunity by promoting the differentiation of Th cells to Treg, which is crucial in reducing post-stroke neuroinflammation (177). Patients with VB_12_ deficiency showed lower levels of Tregs, while VB_12_ supplementation decreased the inflammatory circulating cytokine profiles (177). Furthermore, VB_12_ promotes SCFA-producing commensal bacteria in the gut (178), indicating it might reduce toxic neuroinflammation after IS by promoting gut SCFA (Figure 2). Therefore, in addition to its potential to prevent IS, VB_12_ is also a therapeutic factor in the acute phase of IS.

## Conclusion and future perspectives

In this review, we highlight the role of the gut microbiota and metabolites related to IS. IS results from complex pathophysiological processes, such as oxidative stress, neuroinflammation, and apoptosis. Although several drugs are available to prevent the cascade of events that lead to ischemic injury, limited effective neuroprotective drugs are currently available to improve patient outcomes. In recent years, studies about the relationship between gut microbiota and neurological diseases have become a popular subject, indicating that gut microbiota could also play a vital role in the occurrence, development, and prognosis of IS. Gut microbiota can modulate oxidative stress, inflammation, and apoptosis-related signaling pathways through its constituents (e.g., LPS, peptidoglycan) and metabolites (e.g., SCFAs and BAs). Further, therapies targeting gut microbiota and their metabolites might be promising strategies to prevent or treat IS.

## Author contributions

JL wrote the manuscript. JL, JW, and SC designed the review. JW, YL, and SC revised the manuscript. All authors contributed to the article and approved the submitted version.

## Funding

This study was supported by the Research Funds of Hangzhou Institute for Advanced Study, University of Chinese Academy of Science (B04006C010006 and B04006C018007).

## Conflict of interest

The authors declare that the research was conducted in the absence of any commercial or financial relationships that could be construed as a potential conflict of interest.

## Publisher's note

All claims expressed in this article are solely those of the authors and do not necessarily represent those of their affiliated organizations, or those of the publisher, the editors and the reviewers. Any product that may be evaluated in this article, or claim that may be made by its manufacturer, is not guaranteed or endorsed by the publisher.
